# Comparison of stereotactic radiosurgery and rhizotomy for trigeminal neuralgia: A systematic review and Meta-Analysis

**DOI:** 10.1007/s10143-025-03763-z

**Published:** 2025-08-20

**Authors:** Alireza Soltani Khaboushan, Seyed Farzad Maroufi, Negin Jarrah, Maral Moafi, Mohammadmahdi Sabahi, Hamid Borghei-Razavi, Jason P. Sheehan

**Affiliations:** 1 Department of Neurosurgery, Tehran University of Medical Sciences, Tehran, Iran; 2 School of Medicine, Tehran University of Medical Sciences, Tehran, Iran; 3 Department of Neurosurgery, Johns Hopkins University School of Medicine, Baltimore, MD USA; 4 Cell Biology and Anatomical Sciences, School of Medicine, Shahid Beheshti University of Medical Sciences, Tehran, Iran; 5 Department of Neurological Surgery, Pauline Braathen Neurological Center, Cleveland Clinic Florida, Weston, FL USA; 6 Department of Neurological Surgery, University of Virginia, Charlottesville, VA USA

**Keywords:** Stereotactic radio surgery, Rhizotomy, Trigeminal neuralgia, Balloon compression, Radiofrequency thermoablation, Glycerol rhizotomy

## Abstract

**Objective:**

Trigeminal neuralgia (TN) is a chronic, debilitating neuropathy characterized by sudden, severe facial pain, often refractory to medical therapy. When medications fail, surgical options such as microvascular decompression (MVD) are preferred, but for patients unsuitable for open surgery, stereotactic radiosurgery (SRS) and percutaneous rhizotomy are viable alternatives. This systematic review and meta-analysis aimed to compare the efficacy and safety of SRS and rhizotomy in the management of TN.

**Methods:**

Following PRISMA guidelines, PubMed, Embase, Scopus, and Web of Science were searched up to September 2024 for studies comparing SRS and rhizotomy in TN patients. Eligible studies reported pain relief, recurrence, retreatment rates, or complications. Data were extracted and analyzed using a random-effects model, with subgroup analyses for multiple sclerosis (MS) status.

**Results:**

Fifteen studies involving 1,251 patients (577 SRS, 674 rhizotomy) were included. Rhizotomy provided superior initial pain-free outcomes (RR = 0.66, 95%CI = 0.49 ;0.91, *p* < 0.01), while SRS showed no significant difference in pain-free rates at the last follow-up (RR = 0.99, 95%CI = 0.80 ;1.22, *p* = 0.89) or overall pain relief (RR = 1.14, 95%CI = 0.90 ;1.44, *p* = 0.29). SRS significantly reduced recurrence (RR = 0.70, 95%CI = 0.51 ;0.96, *p* < 0.05), retreatment need (RR = 0.67, 95%CI = 0.46 ;0.96, *p* < 0.05), and facial numbness (RR = 0.61, 95%CI = 0.37 ;0.99, *p* < 0.05). Overall complications were comparable (RR = 0.70, 95%CI = 0.34 ;1.43, *p* = 0.33), though SRS trended toward fewer complications in MS patients.

**Conclusion:**

Rhizotomy provides immediate pain relief, making it suitable for patients requiring rapid results, while SRS offers greater durability and lower morbidity. Treatment choices should be tailored to patient-specific factors, including the urgency of relief and MS status. Future prospective studies with standardized outcomes and extended follow-up are needed to address the limitations of retrospective data and study heterogeneity.

## Introduction

Trigeminal neuralgia (TN) is a chronic severe neuropathy defined by a recurring unilateral or bilateral sudden, brief, electric shock-like pain episode. It occurs in response to triggers affecting regions supplied by single or multiple trigeminal nerve branches. TN could be manifested primarily as classical or idiopathic TN or secondary to an underlying condition like multiple sclerosis (MS) [1]. It is a rare condition with a wide range of incidence from 2.1 to 27 cases per 100,000 person-years that is more prevalent in older patients and women [2, 3].

First-line therapy for patients with TN disorder is medical therapy with anti-epileptic drugs. Surgery is suggested when medical treatment fails and unbearable side effects occur [4]. The most frequent surgical treatment and the primary choice of surgical treatment in refractory TN is microvascular decompression (MVD). MVD is an invasive open craniotomy surgery that is the first line of therapy in classical TN, in which neurovascular compression is evident. However, in the absence of neurovascular compression or in patients who do not tolerate open surgery and struggle with comorbidities, non-invasive treatments are preferred [5]. Rhizotomy and Stereotactic RadioSurgery (SRS) are the alternative treatments that ablate the pathologic nerve. Percutaneous procedures of Rhizotomy are Percutaneous Balloon Compression (PBC), Percutaneous retrogasserian glycerol rhizotomy (PRGR), and Radiofrequency Rhizotomy (RFR). These techniques result in fewer side effects than MVD [6, 7]. SRS is the least invasive technique that delivers a single, high-dose radiation focused on the affected nerve. SRS includes various technologies like Gamma Knife, CyberKnife, and different immobilization methods such as invasive frames and non-invasive masks. The Gamma Knife is often the gold standard for trigeminal neuralgia, with frame-based systems providing superior targeting accuracy. The choice of system affects procedures and outcomes. Gamma knife surgery is the gold standard treatment among SRS methods [8, 9].

High rates of pain relief are reported following all types of rhizotomies. The average period of pain relief is three to four years; a third of the patients are likely to have pain recurrence in a year. The pain-free interval and recurrence rate for different techniques vary among studies [5, 10]. Facial sensory dysesthesia and corneal hypoesthesia are among the complications that may occur following rhizotomy or SRS [11, 12]. Due to the complications and contraindications of open surgery, this review aimed to evaluate and compare the performance of different rhizotomy methods and SRS in treating TN.

## Methods

### Study design and protocol

This systematic review and meta-analysis were conducted to compare the effectiveness and safety of SRS and rhizotomy in the management of TN. The review followed the Preferred Reporting Items for Systematic Reviews and Meta-Analyses (PRISMA) guidelines to ensure transparency and reproducibility. The study protocol has been registered in PROSPERO with the registration ID CRD420251016939.

### Eligibility criteria

Studies were included if they met the following criteria: (1) involved patients diagnosed with TN; (2) compared SRS with rhizotomy methods, including RFR, PRGR, or PBC; (3) reported outcomes related to pain relief or complications, or follow-up data; (4) were original study; and (5) were published in English. Studies were excluded if they were case reports, case series with fewer than 10 patients, reviews, or lacked a direct comparison between SRS and rhizotomy. No restrictions were applied based on the publication date.

### Search strategy

A comprehensive literature search was conducted to identify relevant studies. The databases searched were PubMed, Embase, Scopus, and Web of Science to ensure thorough retrieval of studies. The search spanned from each database’s inception to September 2024, encompassing all available records up to that date. The main keywords used for the search included “trigeminal neuralgia,” “rhizotomy,” “glycerol,” “balloon,” “radiofrequency,” and “radiosurgery.” Search terms were adapted as needed for each database, using Boolean operators to combine terms. Additionally, reference lists of included studies and relevant reviews were manually searched to identify further eligible studies. The full search strategy could be found in the supplementary material 1.

### Study selection

Two reviewers independently screened the titles and abstracts of retrieved records using predefined inclusion and exclusion criteria. Then, full-text articles of potentially eligible studies were retrieved and assessed for inclusion. Reviewer discrepancies were resolved through discussion or consultation with a third reviewer.

### Data extraction

Data were extracted using a standardized form developed for this review. The form captured the following variables: study characteristics (first author, year, country, study design, sample size); patient demographics (total patients, number undergoing SRS or rhizotomy, age, sex, symptom duration); intervention details (type of SRS or rhizotomy and rhizotomy technique); pain outcomes (recurrence rates, time to recurrence, Barrow Neurological Institute [BNI] pain scores); and complications, including facial numbness. For pain outcomes, if the BNI score was provided, a “pain-free” outcome was classified as BNI I, and “pain relief” was classified as BNI I-IIIb. If no BNI score was given, the terminology used in the original paper was taken into account. The follow-up period was different among studies, and outcomes were reported at both initial follow-up (first evaluation following intervention) and last follow-up (last evaluation reported by the study). For more clarity, the median/mean follow-up by studies was also reported. Two reviewers independently extracted data, with discrepancies resolved by consensus or consultation with a third reviewer.

### Risk of bias

The risk of bias of the included studies was assessed using the Joanna Briggs Institute (JBI) Critical Appraisal Checklist for Quasi-Experimental Studies. The checklist consists of nine questions evaluating aspects such as clarity of cause and effect, similarity of participants across groups, presence of a control group, consistency of outcome measurement, and appropriateness of statistical analysis. Each question was scored as “Yes,” “No,” “Unclear,” or “Not Applicable” by two independent reviewers.

### Data synthesis and analysis

Where sufficient data were available and heterogeneity permitted, a meta-analysis was conducted to pool effect sizes for key outcomes. Dichotomous outcomes were analyzed using risk ratios (RR) with 95% confidence intervals (CI). A random-effects model was used to address expected clinical and methodological diversity among studies. The I² statistic and the Cochrane Q test evaluated statistical heterogeneity. A p-value above 0.1 indicates significance. Heterogeneity levels are categorized as low (*I*^*2*^ < 25%), moderate (*I*^*2*^ < 25 ;75%), and high (*I*^2^ > 75%). Subgroup analyses differentiated between patients with MS and those without. Sensitivity analyses were conducted to assess the robustness of findings by excluding studies with a high risk of bias. All statistical analyses were performed using R version 4.4.0 with a significance level of *p* < 0.05.

## Results

### Study selection

**Fig. 1:**
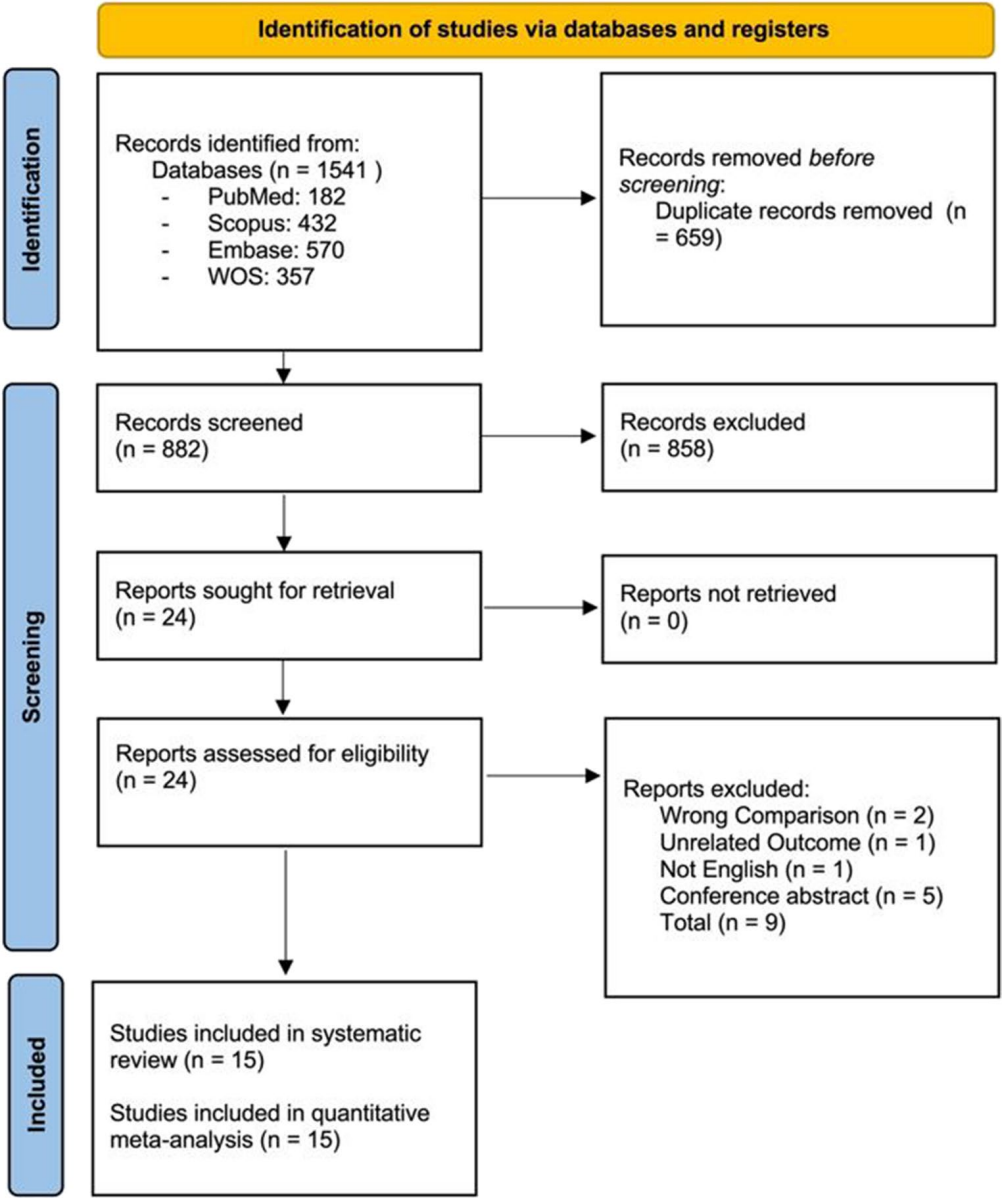
PRISMA Flow Diagram of Study Selection Process

A total of 1,541 references retrieved. After removing 659 duplicates, 882 articles remained for screening. During the title and abstract screening phase, 858 articles were excluded based on our eligibility criteria, leaving 24 articles for full-text review [13–36]. Upon full-text review, 9 articles were excluded [28–36]. Consequently, 15 articles remained for meta-analysis [13–27]. Out of 9 papers excluded in full-text screening, two did not report a comparison of interest [31, 36]one was not English [35]one had an unrelated outcome [29]and five were abstracts [28, 30, 32–34]. Figure 1 presents the PRISMA flow diagram that outlines the process of article selection and screening.

### Study characteristics and risk of bias

The meta-analysis included 15 studies conducted between 2005 and 2023, spanning multiple countries: the USA (*n* = 10), Canada (*n* = 2), Korea (*n* = 1), Italy (*n* = 1), and Mexico (*n* = 1). These studies collectively assessed 1,251 patients with TN, with 577 receiving SRS and 674 undergoing rhizotomy procedures, including RFR, PBC, and PRGR. Patient mean/median ages ranged from 50 to 75 years, and sample sizes varied widely from 11 to 327 participants. Eight studies included patients with MS (1 had both MS and non-MS), while seven focused exclusively on non-MS populations. Treatment strategies were classified as upfront interventions (*n* = 5), retreatment following prior procedures (*n*= 2), or a mixed approach (*n* = 8). SRS radiation doses, where specified, ranged from 40 Gy (usually for retreatment) to 90 Gy (for upfront treatment). Pain relief outcomes showed considerable variation, with SRS achieving initial pain relief in 33.3 ;100% of cases and rhizotomy in 33.3 ;83.3%, though some studies omitted specific outcome data. Regarding the follow-up, the weighted mean showed a mean follow-up of 42.67 months for SRS and 42.25 months for Rhizotomy, which were comparable (*p* = 0.88). Table 1 provides a comprehensive overview of these characteristics.

**Table 1:** Study characteristics of included studies

First Author	Year	Country	Patients (SRS/Rhiz)	Age (years) (SRS/Rhiz)	SRS Dose	Rhizotomy Detail	Follow-up (SRS/Rhiz)	MS	Treatment Approach	Initial Pain Outcome (SRS; Rhiz)
Sanchez-Mejia	2005	USA	20/6	Total: 69.1	40 Gy	RFR	28/30	No	Retreat	Initial: NR; Last outcome: 8/20 (40%); 4/6 (66.7%)
Pollock	2010	USA	24/16	68/66.8	80 ;85 Gy	RFR, BMC, PRGR	Total: 35	No	Mixed	16/24 (66.7%); 10/16 (62.5%)
Pollock	2005	USA	69/51	67.8/72.2	70 ;90 Gy	PRGR	Total: 20.6	No	Mixed	45/69 (65.2%); 35/51 (68.6%)
Mohammad-Mohammad	2013	USA	52/186	Total: 50	75 ;86 Gy	PBC, PRGR, RFR	Total: 69	Yes	Upfront	12/24 (50%); 52/65 (80%)
									Retreat	17/28 (60.7%); 86/121 (71%)
Mathieu	2012	Canada	27/18	59/56.5	80 ;90 Gy	PRGR	39/38	Yes	Upfront	19/27 (70.4%); 15/18 (83.3%)
Lee	2020	USA	23/19	61/57	80 ;85 Gy	RFR	96/42	Yes	Upfront	9/18 (50%); 12/16 (75%)
Han	2010	Korea	31/161	NR/NR	70 ;90 Gy	RFR, PRGR	Total: 56.4	No	Upfront	14/31 (45.2%); 129/161 (80.1%)
Alvarez-Pinzon	2016	USA	124/78	51/53	37 Gy	PBC	Total: 37.6	Yes	Upfront	97/124 (78.2%); 31/78 (39.7%)
Cheng	2005	USA	6/5	Total: 69	70 ;85 Gy	RFR	Total: 40.6	Yes	Mixed	Initial: NR; Last outcome: 4/4 (100%); 2/6 (33.3%)
Hitchon	2016	USA	80/36	73/75	90 Gy	RFR	Total: 32	No	Upfront	NR; NR
Holland	2017	USA	7/10	58.5/63.5	90 Gy	RFR	66/10.4	Yes	Upfront	NR; NR
Ferraro	2019	Italy	30/33	Total: 58	NR	RFR, PRGR, PBC	Total: 60	Yes	Mixed	NR; NR
Krishnan	2017	Canada	12/9	56/57	80 Gy	PRGR, PBC	38/126	Yes	Mixed	NR; NR
Altamirano	2023	Mexico	9/10	62.6/61.6	85 Gy	RFR	33.86/43.91	No	Retreat	3/9 (33.3%); 4/9 (44.4%)
Hensen	2005	USA	63/36	70/69	70 ;90 Gy	PRGR	29/34	NR	Mixed	28/63 (44.4%); 21/36 (58.3%)

*SRS* Stereotactic Radiosurgery, *Rhiz* Rhizotomy, *MS *Multiple Sclerosis, *RFR *Radiofrequency Rhizotomy, *PRGR *Percutaneous Retrogasserian Glycerol Rhizotomy, *BMC* Balloon Microcompression, *PBC *Percutaneous Balloon Compression, *NR *Not Reported

Risk of bias scores ranging from 33.33 to 100%. Altamirano (2023) [13] scored a perfect 100%, reflecting high methodological rigor, while Ferraro (2019) [16] scored the lowest at 33.33%, indicating significant limitations. Most studies [14, 15, 17–27] (66.67 ;77.78%) demonstrated moderate to good quality. Table 2 provides a summary of the risk of bias.

**Table 2:** Risk of bias assessment of included studies

Author, Year	1	2	3	4	5	6	7	8	9	Overall %
Cheng, 2005	Yes	No	Yes	Yes	No	No	Yes	Yes	Yes	66.67
Altamirano, 2023	Yes	Yes	Yes	Yes	Yes	Yes	Yes	Yes	Yes	100
Alvarez-Pinzon, 2017	Yes	No	Yes	Yes	No	Yes	Yes	Yes	Yes	77.78
Han, 2010	Yes	No	Yes	Yes	No	Yes	Yes	Yes	Yes	77.78
Henson, 2005	Yes	Unclear	Yes	Yes	Yes	No	Yes	Yes	Yes	77.78
Hitchon, 2016	Yes	Yes	Yes	Yes	Unclear	No	Yes	Unclear	Yes	66.67
Holland, 2017	Yes	Yes	Yes	Yes	No	Unclear	Yes	Unclear	No	55.56
Ferraro, 2019	Yes	Unclear	No	Yes	No	Unclear	Yes	Unclear	No	33.33
Krishnan, 2017	Yes	Yes	No	Yes	No	Unclear	Yes	No	Yes	55.56
Lee, 2020	Yes	Yes	Yes	Yes	No	No	Yes	Yes	Yes	77.78
Pollock, 2010	Yes	No	Yes	Yes	No	No	Yes	Yes	Yes	66.67
Pollock, 2005	Yes	No	Yes	Yes	No	Unclear	Yes	Yes	Yes	66.67
Sanches-Mejia, 2005	Yes	No	Yes	Yes	No	No	Yes	Yes	Yes	66.67
Mathieu, 2012	Yes	Yes	Yes	Yes	No	No	Yes	Yes	Yes	77.78
mohammad-mohammadi, 2013	Yes	Unclear	Yes	Yes	No	No	Yes	Yes	Yes	66.67

Questions: 1. Is it clear in the study what is the ‘cause’ and what is the ‘effect’? 2. Were the participants included in any comparisons similar? 3. Were the participants included in any comparisons receiving similar treatment/care, other than the exposure or intervention of interest? 4. Was there a control group? 5. Were there multiple measurements of the outcome, both pre- and post-intervention/exposure? 6. Was follow-up complete and if not, were differences between groups adequately described and analyzed? 7. Were the outcomes of participants included in any comparisons measured in the same way? 8. Were outcomes measured in a reliable way? 9. Was appropriate statistical analysis used?

### Pain-free outcome at last follow-up

**Fig. 2:**
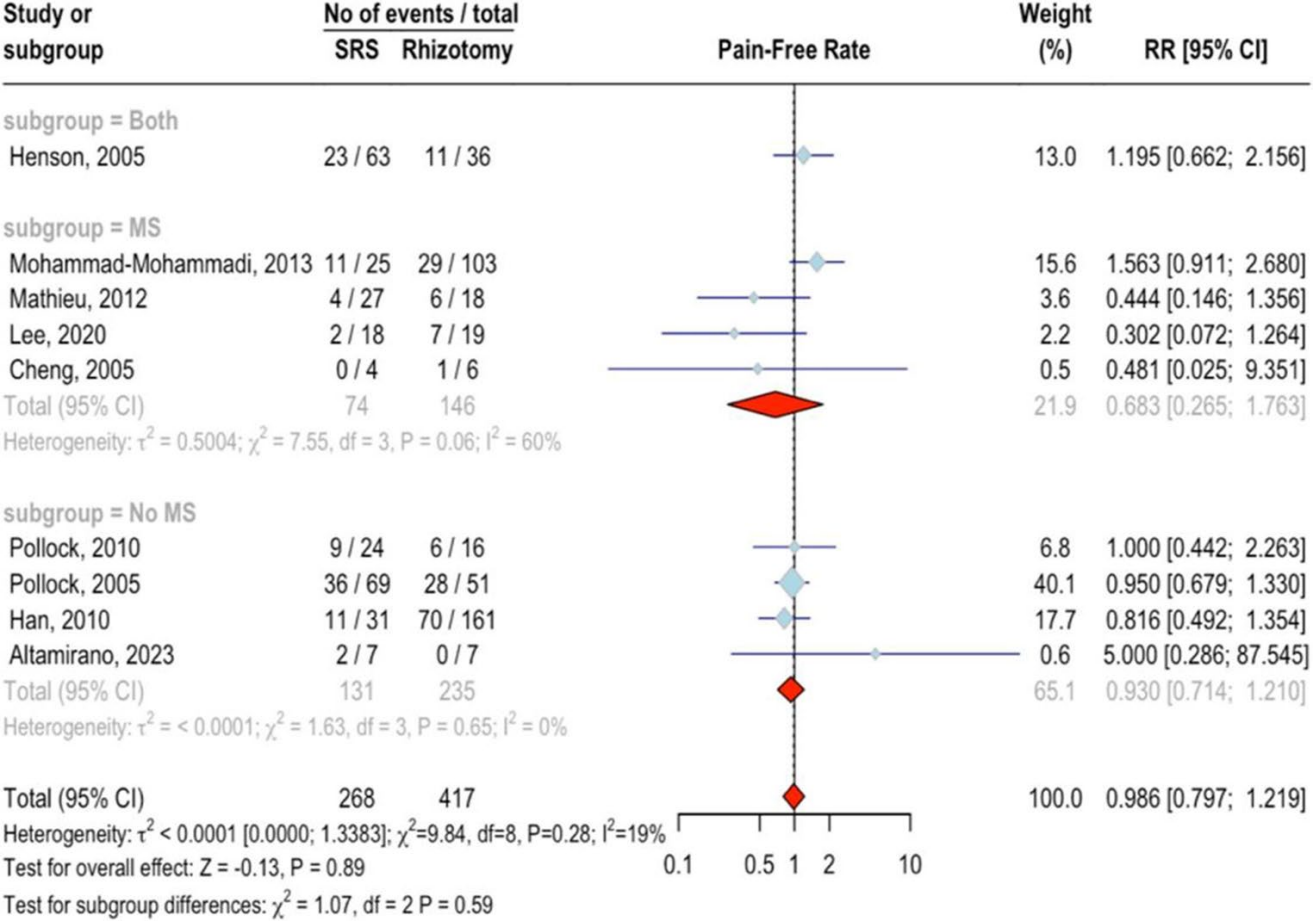
Forest plot for the meta-analysis of pain-free outcome at the last follow-up

Nine studies involving 685 patients were analyzed, with 268 patients receiving SRS and 417 undergoing rhizotomy, resulting in 256 events being pain-free at the last follow-up. Using a random effects model, the RR was calculated as 0.99 (95%CI = 0.80 ;1.22, *p* = 0.9771), indicating no significant difference in efficacy between SRS and rhizotomy (Fig. 2). The studies showed low heterogeneity, as evidenced by an I² of 19%, with a non-significant test for heterogeneity (*Q* = 9.84, *p* = 0.28). Subgroup analysis based on MS status further supported this finding: one study including both MS and non-MS patients reported an RR of 1.20 (95%CI = 0.66 ;2.16); four studies with MS patients showed an RR of 0.68 (95%CI = 0.27 ;1.76, I²=40.1%); and four studies with non-MS patients had an RR of 0.93 (95%CI = 0.71 ;1.21, I²=0%). The test for subgroup differences was not significant (*p* = 0.59). No outliers were identified, and there was no evidence of publication bias (*p* = 0.61). Meta-regression analyses showed that the percentage of female patients (*p* = 0.24) and age (*p* = 0.30) did not significantly impact the treatment effect. Additionally, upfront treatment was linked to poorer outcomes in SRS group (*p* < 0.05).

### Initial pain-free outcome

**Fig. 3:**
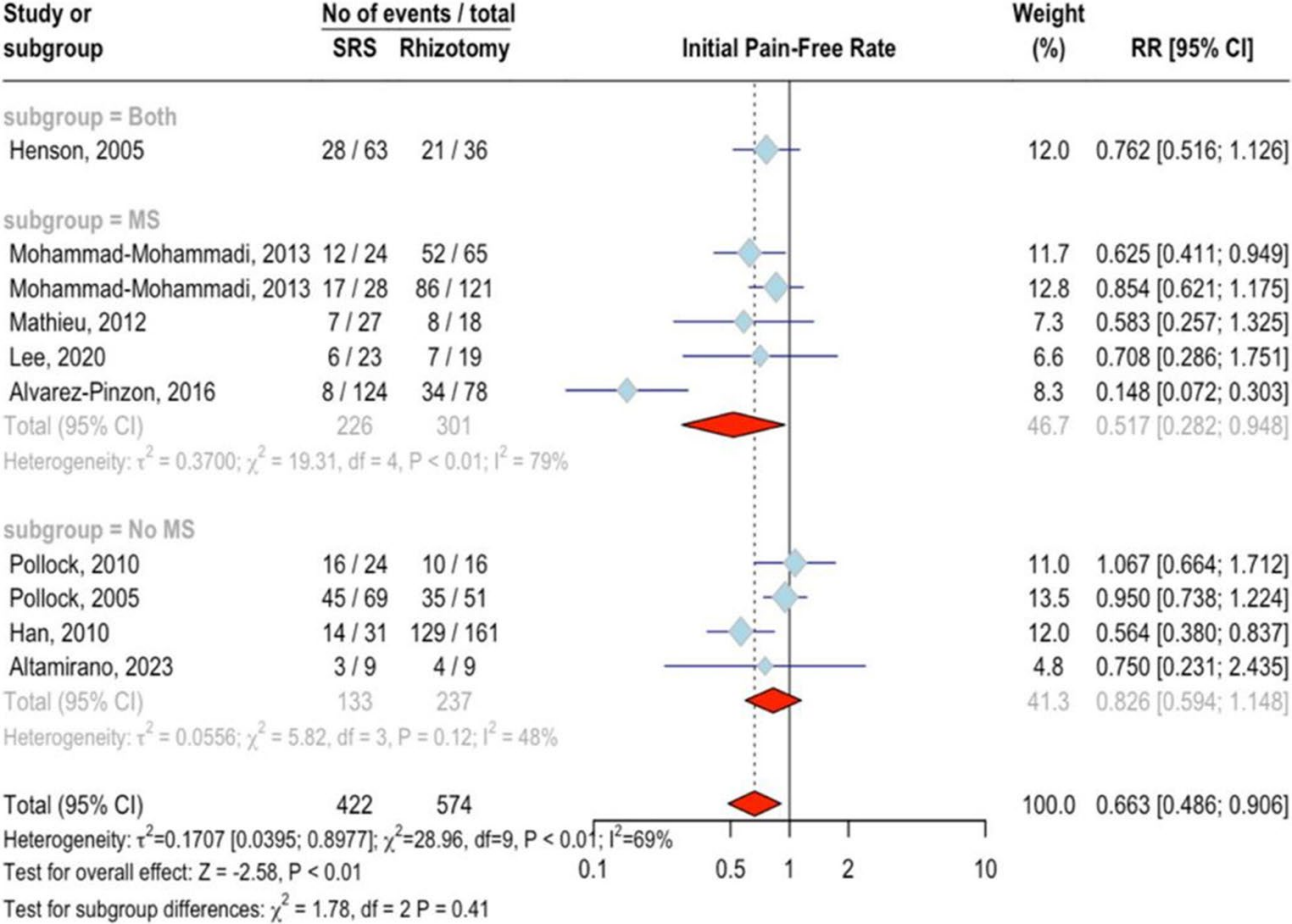
Forest plot for initial pain-free outcome meta-analysis

Nine studies with 996 patients were included, comprising 422 patients treated with SRS and 574 with rhizotomy, resulting in 542 events being pain-free immediately post-treatment. The analysis showed that SRS was significantly less effective than rhizotomy, with an RR of 0.66 (95%CI = 0.49 ;0.91, *p* < 0.01; Fig. 3). Substantial heterogeneity was observed, with an I² of 68.9% (Q = 28.96, *p* < 0.001). Subgroup analysis by MS status indicated an RR of 0.76 (95%CI = 0.52 ;1.13) for one study with both MS and non-MS patients, an RR of 0.52 (95%CI = 0.28 ;0.95, I²=79.3%) for five effects of MS patients, and an RR of 0.83 (95%CI = 0.59 ;1.15, I²=48.4%) for four studies with non-MS patients; however, the test for subgroup differences was not significant (Q = 1.78, *p* = 0.41). One outlier, identified as “Alvarez-Pinzon, 2016,” was detected. Upon its removal, the RR increased to 0.79 (95%CI = 0.66 ;0.93, *p* < 0.01), and heterogeneity decreased markedly to an I² of 5.0% (Q = 8.42, *p* = 0.39). No evidence of publication bias was found (*p* = 0.15). Meta-regression analyses indicated no significant effects from the percentage of female patients (*p* = 0.25) or age (*p* = 0.26); however, the type of treatment showed a significant effect, with upfront treatment linked to worse outcomes in the SRS group (*p* < 0.05), though the overall test for treatment type was not significant (*p* = 0.16).

###  Pain-relief outcome

**Fig. 4:**
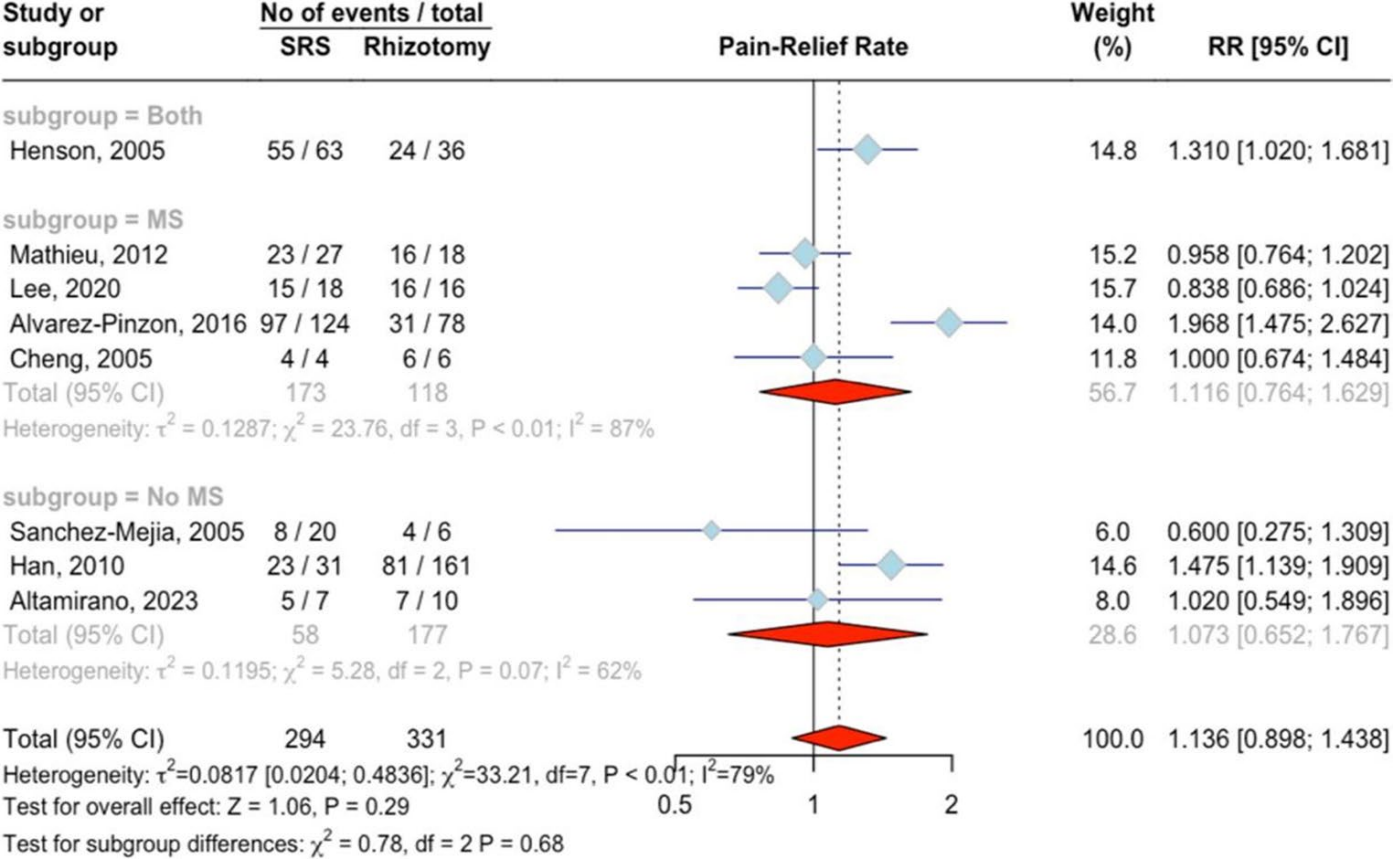
Forest plot for the meta-analysis of pain-relief outcome at last follow-up

Eight studies involving 625 patients were analyzed, with 294 patients treated with SRS and 331 with rhizotomy, resulting in 289 events of pain relief (BNI I-IIIb) at the last follow-up. The RR was 1.14 (95%CI = 0.90 ;1.44, *p* = 0.29; Fig. 4), indicating no significant difference between the two treatments. High heterogeneity was present, with an I² of 78.9% and the test for heterogeneity was significant (Q = 26.16, *p* < 0.001). Subgroup analysis by MS status showed an RR of 1.31 (95%CI = 1.02 ;1.68) for one study with both MS and non-MS patients, an RR of 1.16 (95%CI = 0.76 ;1.63, I²=86.7%) for four studies with MS patients, and an RR of 1.07 (95%CI = 0.65 ;1.77, I²=62.1%) for three studies with non-MS patients, with a non-significant test for subgroup differences (Q = 0.78, *p* = 0.68). “Alvarez-Pinzon, 2016” was identified as an outlier, and its removal resulted in an RR of 1.05 (*p* = 0.62) with I [2] of 65.1% (Q = 17.20, *p* < 0.01). There was no evidence of publication bias (*p* = 0.98). Meta-regression analyses showed no significant effects for the percentage of female patients (*p* = 0.87), or type of treatment (*p* = 0.70), and the analysis for age could not be conducted due to insufficient data.

### Need for retreatment 

**Fig. 5:**
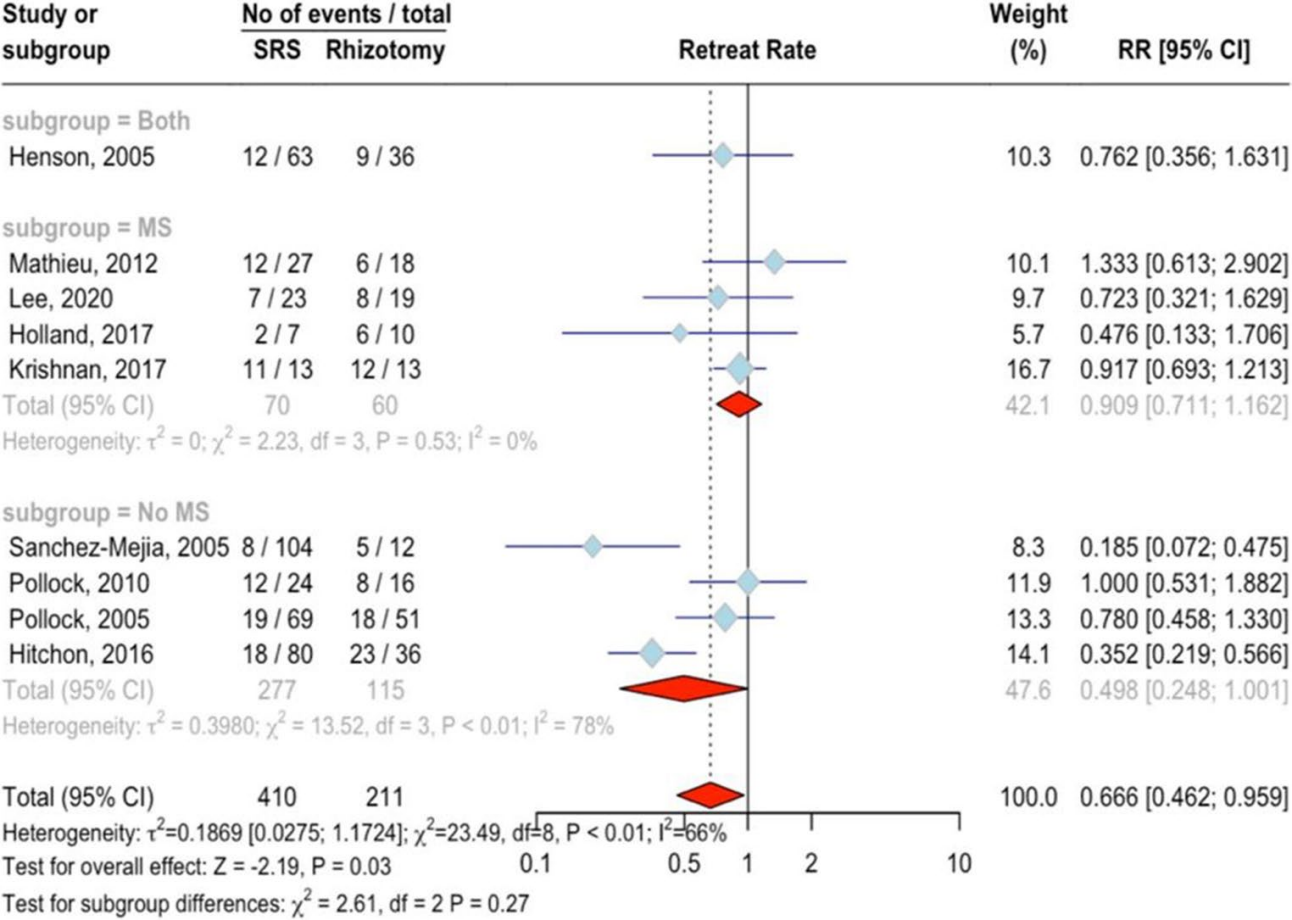
Forest plot for the retreat rate meta-analysis

Data from nine studies involving 621 patients (410 treated with SRS and 211 with rhizotomy) showed that SRS significantly reduced the need for additional interventions compared to rhizotomy with an RR of 0.67 (95%CI = 0.46 ;0.96, *p* = 0.03) (Fig. 5). There was moderate variation across studies (I²=65.9%, *p* < 0.01), but no evidence of publication bias (*p* = 0.39). No significant differences were observed between MS (RR = 0.91, 95%CI = 0.71 ;1.16, I^2^ = 0.0%) and non-MS group (RR = 0.50, 95%CI = 0.25 ;1.00, I^2^ = 77.8%, *p* = 0.27). No outliers were identified in the studies. Meta-regression revealed that SRS as a retreatment was associated with a reduced need for further procedures (*p* < 0.05). Neither the percentage of female patients (*p* = 0.60) nor age (*p* = 0.48) significantly affected this outcome

### Recurrence rate

**Fig. 6:**
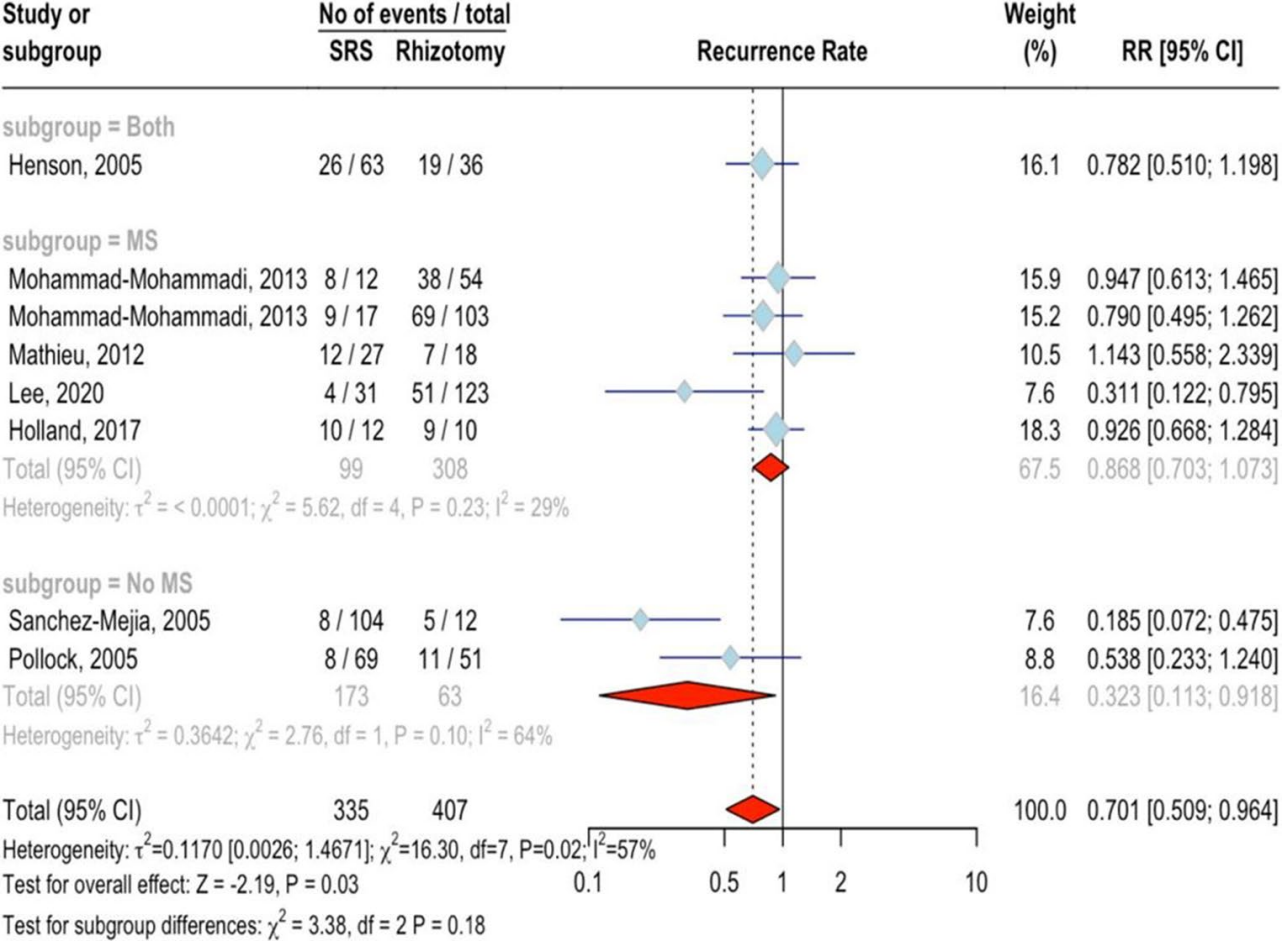
Forest plot for recurrence rate meta-analysis

Seven studies with 742 patients (335 SRS, 407 rhizotomy) were examined. SRS was associated with a lower recurrence rate than rhizotomy, with an RR of 0.70 (95%CI = 0.51 ;0.96, *p* < 0.05; Fig. 6). Moderate heterogeneity was present (I²=57.1%, *p* < 0.05), and there was evidence of publication bias (*p* < 0.05), suggesting the possibility that the treatment effect might be overestimated. Trim-and-fill with three added studies showed an RR of 0.90 (95%CI = 0.57 ;1.40; *p* = 0.63). No significant differences were found between MS (RR = 0.87, 95%CI = 0.70 ;1.07, I^2^ = 28.8%) and non-MS groups (RR = 0.32, 95% CI = 0.11 ;0.92, I^2^ = 63.8%, *p* = 0.18). “Sanchez-Mejia, 2005” was identified as an outlier, and its removal made the result non-significant (RR = 0.83, 95%CI = 0.69 ;1.00; *p* = 0.051). Notably, older age was associated with a lower recurrence rate in SRS (*p* < 0.01), while the percentage of female patients (*p* = 0.15) and treatment type (*p* = 0.51) showed no significant impact

### Complications

#### Facial numbness

**Fig. 7:**
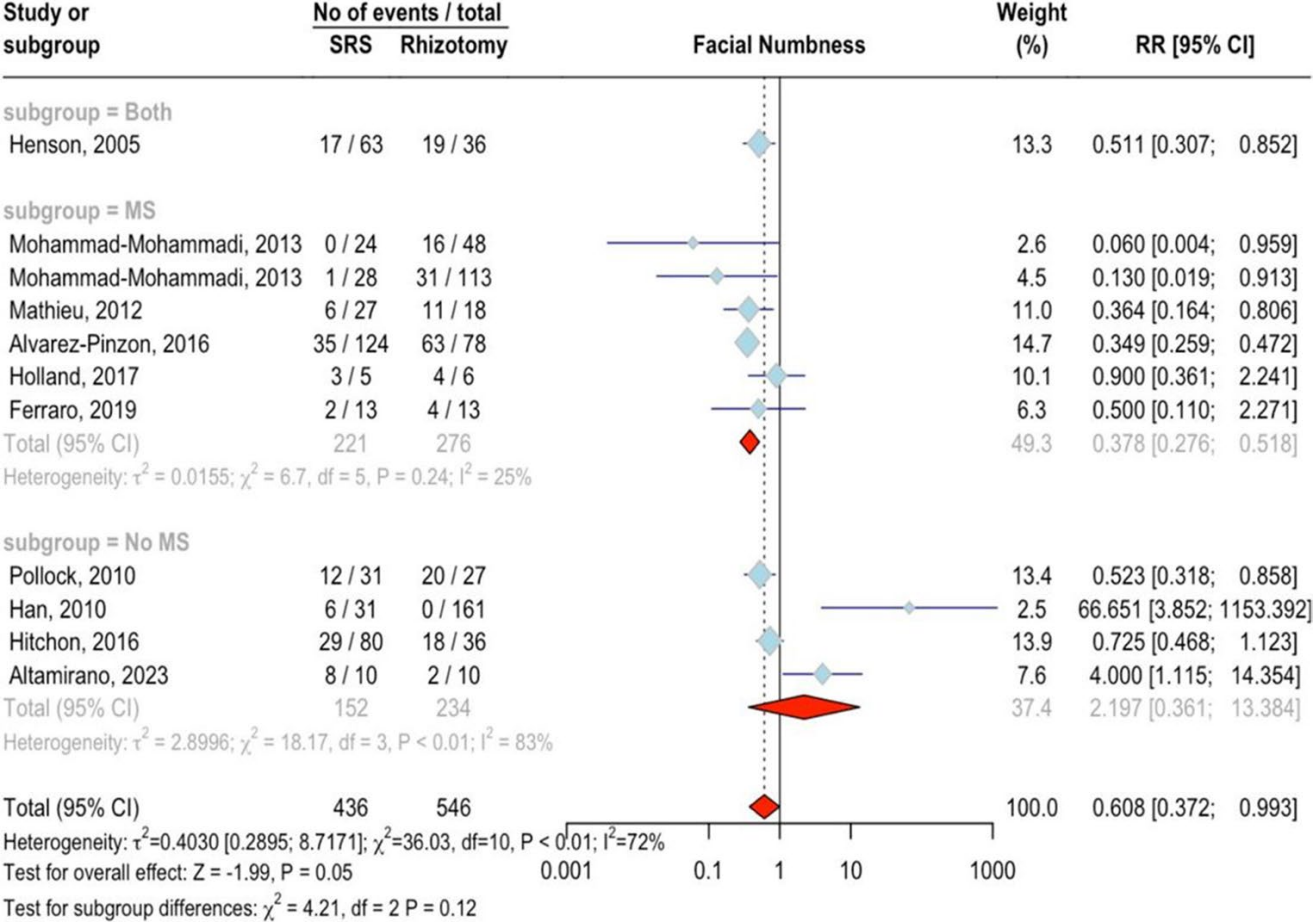
Forest plot for facial numbness outcome meta-analysis

Ten studies involving 982 patients (436 SRS, 546 rhizotomy) were analyzed. SRS significantly reduced the risk of facial numbness compared to rhizotomy, with an RR of 0.61 (95%CI = 0.37 ;0.99, *p* < 0.05; Fig. 7). There was no significant difference between MS (RR = 0.38, 95%CI = 0.28 ;0.52, I^2^ = 25.4%) and non-MS groups (RR = 2.20, 95%CI = 0.36 ;13.38, I^2^ = 83.5, *p* = 0.12). Initially, there was high variation between studies (I²=72.2%, *p* < 0.0001), but this decreased after removing two outlier studies (“Han, 2010” and “Altamirano, 2023”), resulting in a stronger effect (RR = 0.48, *p* < 0.0001, I²=42.4%). No publication bias was detected (*p* = 0.27). A higher percentage of female patients was associated with a lower risk of facial numbness in the SRS group (*p* < 0.01), while age had a borderline effect (*p* = 0.09). The impact of the treatment type was notable yet not clearly defined (*p* < 0.01). The study “Ferraro 2019” had a high risk of bias, but removing it from this analysis did not lead to any significant changes in the overall effect [16]

#### Overall Complications

**Fig. 8:**
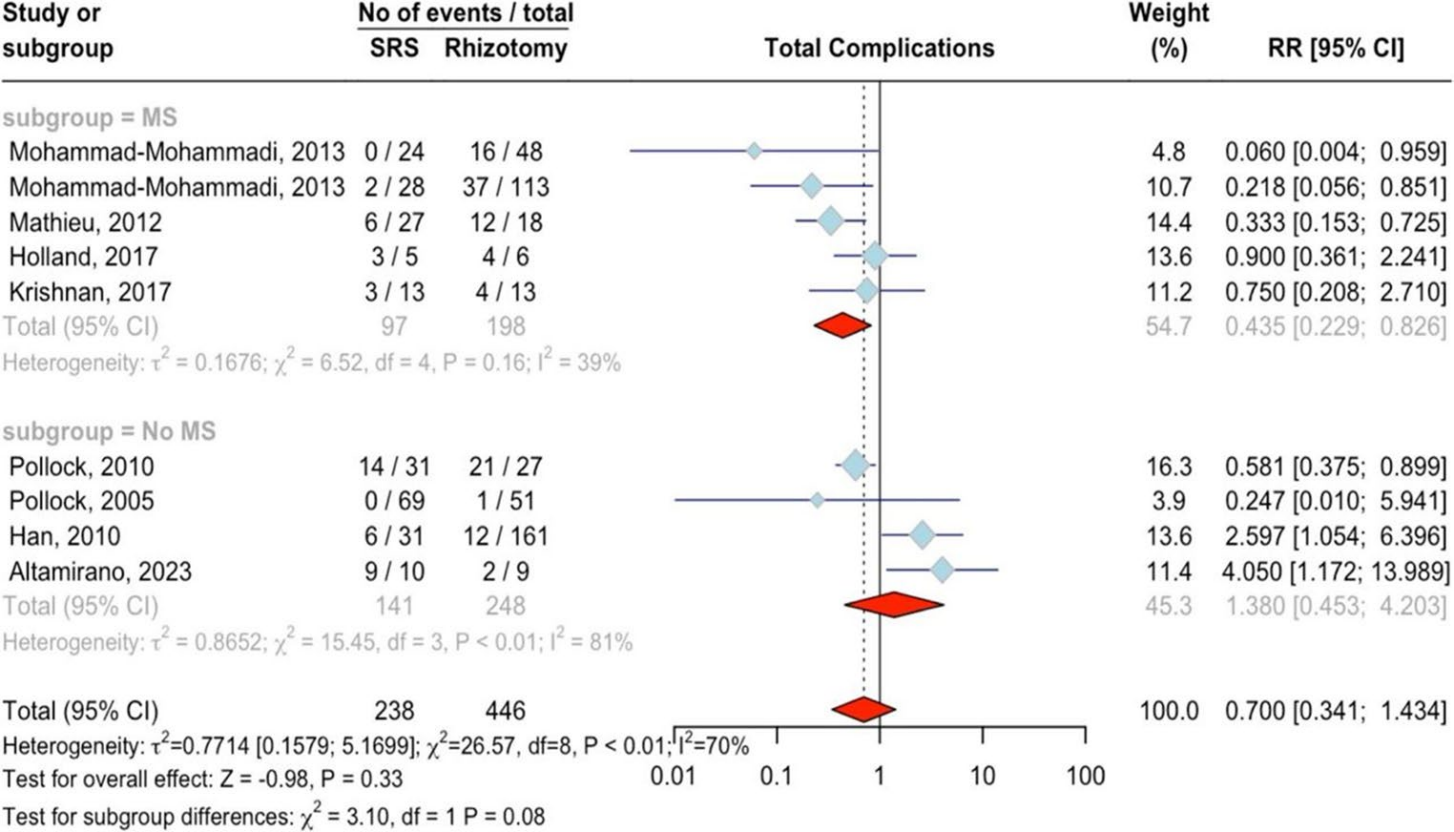
Forest plot for the total complication outcome meta-analysis

Eight studies with 684 patients (238 SRS, 446 rhizotomy) were included. There was no significant difference between SRS and rhizotomy, with an RR of 0.70 (95%CI = 0.34 ;1.43, *p* = 0.33; Fig. 8). High heterogeneity was observed (I²=69.9%, *p* < 0.001), but no outliers or publication bias were found (*p* = 0.93). No significant difference was observed between MS (RR = 0.44, 95%CI = 0.23 ;0.83, I^2^ = 38.6%) and non-MS subgroups (RR = 1.38, 95%CI = 0.45 ;4.20, I^2^ = 80.6%, *p* = 0.08). The publication bias of the studies was not significant (*p* = 0.93). A higher percentage of female patients was significantly associated with fewer complications in the SRS group (*p* < 0.01), while age (*p* = 0.35) and treatment type (*p* = 0.51) had no notable influence. Table 3 reports a summary of meta-regression results Complications varied between rhizotomy and SRS, both of which were clinically significant. Transient complications, especially in SRS patients, included facial numbness, sensory issues, and dysesthesia, which generally resolved in weeks to months. Though temporary, they caused discomfort but had little long-term impact [37, 38]. Rhizotomy procedures caused mild to moderate facial pain, dysesthesia, or transient weakness. Most complications were treatable with short-term corticosteroids or analgesics. While mostly reversible, they temporarily reduced quality of life and sometimes required follow-up visits or treatments [39, 40]. Rhizotomy was associated with a greater risk of severe, long-term sequelae, including permanent facial hypoesthesia, anesthesia dolorosa, and a frequent need for retreatment. In contrast, while SRS also carried risks of chronic sensory disturbances, particularly at higher radiation doses, its profile was less hazardous as severe, permanent complications were less common, resulting in a lower overall clinical burden [26, 27, 41, 42]

**Table 3:** Summary of Meta-Regression results

Outcome	Moderator	Number of Effects (k)	Estimate (Coefficient)	*p*-value	*R*² (% Variability Explained)	Residual Heterogeneity (I²)
Pain-Free at Last Follow-Up	Female	8	−2.4695	0.2427	0.00%	39.62%
	Age	3	−0.0304	0.2968	0.00%	0.00%
	Type of Tx	9	Mixed: −0.2014, Retreat: 0.4881, Upfront: −0.9555	0.4928, 0.1087, 0.0422^*^	100%	0.00%
Initial Pain-Free	Female	9	−1.5479	0.2505	17.41%	75.84%
	Age	3	0.0211	0.2582	0.00%	26.27%
	Type of Tx	10	Mixed: −0.4840, Retreat: −0.0989, Upfront: −0.7195	0.2973, 0.8086, 0.0335^*^	28.43%	64.23%
Pain Relief	Female	7	0.1273	0.8690	0.00%	81.73%
	Age	-	-	-	-	-
	Type of Tx	8	Mixed: 0.2428, Retreat: −0.3603, Upfront: −0.0058	0.6008, 0.4198, 0.9868	0.00%	84.79%
Need for Retreatment	Female	9	−1.4142	0.5981	0.00%	69.98%
	Age	3	−0.0610	0.4791	0.00%	86.91%
	Type of Tx	9	Retreat: −1.5179, Upfront: −0.1943	0.0202^*^, 0.5771	35.88%	56.56%
Recurrence Rate	Female	8	−3.3514	0.1518	34.08%	50.93%
	Age	3	−0.0812	0.0023^**^	100.00%	0.00%
	Type of Tx	8	Retreat: −0.3867, Upfront: 0.1835	0.4923, 0.7004	0.00%	70.29%
Facial Numbness	Female	10	−3.6246	0.0018^**^	79.22%	22.19%
	Age	4	0.0806	0.0869	100.00%	0.00%
	Type of Tx	11	Mixed: 4.8635, Retreat: 0.9005, Upfront: −0.0640	0.0015^**^, 0.1919, 0.8698	62.80%	57.04%
Overall Complications	Female	8	−4.9680	0.0017^**^	97.31%	5.53%
	Age	4	0.0500	0.3543	0.00%	31.23%
	Type of Tx	9	Mixed: 1.6893, Retreat: 0.7150, Upfront: −0.0648	0.2350, 0.5636, 0.9525	0.00%	70.43%

## Discussion

The management of TN often involves a nuanced comparison between SRS and percutaneous rhizotomy, two ablative modalities with distinct mechanisms, outcomes, and risks. SRS delivers high-dose radiation (70 ;90 Gy) to the trigeminal nerve’s cisternal segment, inducing delayed axonal degeneration of nociceptive fibers over weeks to months. This radiobiological effect correlates with pain relief but also carries a 20 ;30% risk of facial hypoesthesia, which paradoxically predicts better long-term outcomes [43, 44]. In contrast, rhizotomy—whether via RFR, PRGR, or PBC—achieves immediate pain relief through direct nerve ablation. While rhizotomy boasts > 95% initial success, its durability is limited, with recurrence rates exceeding 50% at 3 years due to nerve regeneration. Additionally, rhizotomy is associated with higher rates of bothersome sensory deficits (40 ;60% hypoesthesia, 1 ;5% anesthesia dolorosa) and procedural risks such as corneal anesthesia or masseter weakness [10, 45].

This meta-analysis evaluated the efficacy and safety of SRS versus rhizotomy for TN. We assessed pain outcomes, retreatment and recurrence rates, and complications with subgroup analyses to explore differences in patients with and without MS. Both treatments provide comparable pain relief, with no significant difference in pain-free rates at last follow-up (RR = 0.99, *p* = 0.89) or overall pain relief (RR = 1.14, *p* = 0.29), though rhizotomy offers superior initial pain-free outcomes (RR = 0.66, *p* < 0.01). SRS significantly reduces the need for retreatment (RR = 0.67, *p* < 0.05) and recurrence rates (RR = 0.70, *p* < 0.05). Additionally, SRS is associated with a lower risk of facial numbness (RR = 0.61, *p* < 0.05). Overall complication rates showed no significant difference (RR = 0.70, *p* = 0.33), though MS patients may experience fewer complications with SRS than with rhizotomy. These findings highlight SRS’s durability and safety advantages, balanced against rhizotomy’s immediate efficacy, informing tailored TN management.

### Patient selection

Patient selection plays a pivotal role in optimizing outcomes. SRS is favored for medically frail patients or those prioritizing minimal invasiveness, particularly when neurovascular conflict precludes microvascular decompression (MVD) [46]. Its delayed efficacy (85 ;90% pain relief at 1 year) and low acute morbidity make it suitable for individuals willing to tolerate a latency period [47]. Rhizotomy, however, is ideal for patients requiring immediate relief, such as those in acute crisis or those with contraindications to radiation [48]. Salvage strategies further differentiate these approaches: SRS recurrence may be managed with repeat SRS (50 Gy) or MVD, though prior radiation complicates posterior fossa exploration due to arachnoid scarring [49]. Post-rhizotomy recurrence often necessitates MVD or SRS, but prior ablative procedures heighten hypoesthesia risks.

Unlike classical TN, in patients with MS, demyelinating plaques at the trigeminal nerve root are the main cause of symptoms. In addition, central demyelinating lesions within the trigeminal nucleus may contribute independently to TN. Therefore, patients presenting with TN who do not exhibit neurovascular compression are often diagnosed with MS and have demyelinating plaques. In some cases, simultaneous neurovascular compression coexists, which can worsen demyelination by adding mechanical pressure and promoting inflammation [50, 51]. As a result, Rhizotomy and particularly SRS, which ablate the nerve root, are usually preferred in patients with MS. SRS uses concentrated radiation close to the nerve root, blocking the pain signal pathways. It could target different distances from the root entry zone, either proximally, offering stronger pain relief at the cost of higher risk of facial numbness, or distally at the cisternal segment, which may lead to fewer complications. However, percutaneous ablations affecting the trigeminal ganglion are less selective in targeting specific divisions of the trigeminal nerve [50–52].

Technical nuances also influence decision-making. SRS requires precise dose selection (80 ;90 Gy) to balance efficacy and hypoesthesia risk, with frame-based systems offering superior targeting accuracy. Rhizotomy demands meticulous foramen ovale cannulation and intraoperative stimulation to minimize complications. Ultimately, while MVD remains the gold standard for durable pain control without sensory loss, SRS and rhizotomy serve critical roles in high-risk or recurrent cases. A multidisciplinary approach, integrating patient priorities, anatomical factors, and prior treatments, is essential to tailor therapy effectively [9, 53, 54].

### Pain outcomes

The treatment of TN with rhizotomy and SRS offers distinct pain outcomes, shaped by their differing mechanisms, efficacy profiles, and complication risks. Rhizotomy provides immediate pain relief by selectively ablating nociceptive fibers in the trigeminal ganglion via thermocoagulation. Approximately 70 ;90% of patients achieve complete pain relief immediately post-procedure, though durability is limited, with median pain-free intervals of 12 ;24 months and recurrence rates of 30 ;50% at 3 ;5 years [10, 55–58]. Higher lesioning temperatures (> 70 °C) and longer ablation durations (> 90 s) correlate with prolonged relief but increase risks of facial hypoesthesia (15 ;30%) and dysesthesia (5 ;10%) [59, 60]. In contrast, SRS delivers focused radiation (70 ;90 Gy) to the cisternal trigeminal nerve, inducing delayed neuromodulatory effects over weeks to months. While 70 ;90% of patients achieve significant pain relief within 3 ;8 weeks, durability is superior to rhizotomy, with 50 ;60% maintaining relief at 3 years and 30 ;40% at 5 years [61, 62]. It should be noted that dose escalation (≥ 90 Gy) may enhance the efficacy of SRS at the cost of higher hypoesthesia rates (20 ;30%) [63].

In terms of comparing SRS and rhizotomy, our meta-analysis revealed no significant difference in pain-free rates at either follow-up or initial assessment or in overall pain relief rates between SRS and the combined rhizotomy group. This finding is consistent with several studies. Lee et al. [22] reported similar initial pain freedom outcomes between SRS and RFR in MS-related TN. Holland et al. [20] found that both SRS and RFR provided good initial pain relief for TN in the MS setting. Mathieu et al. [23] conducted a comparative study of GKS and PRGR for MS-related TN, aiming to evaluate clinical outcomes, though they did not specifically report pain-free rates. Some studies suggest differences in the duration of pain relief, which our meta-analysis may not have fully captured. Lee et al. [22] indicated that SRS patients with pain relief had longer intervals to pain recurrence at two years compared to RFR in MS-TN. Mohammad-Mohammadi et al. [24] found that PBC, as an initial procedure for MS-related TN, had the highest initial pain-free response and median pain-free interval compared to glycerol rhizotomy and SRS. Moreover, in a study by Alvarez-Pinzon et al. [14] the initial pain-free rate was significantly lower in the SRS group compared to the PBC group, which was identified as an outlier in our analysis. This discrepancy may be attributed to the use of a dosage of only 37 Gy (50% of the isodose line) despite it being an upfront treatment. Additionally, PBC is recognized as one of the rhizotomy methods with the highest initial pain-free rate. Furthermore, the study lacks a clear definition of what constitutes the initial pain-free outcome, which may contribute to this finding. This may be due to the variations in the definition and time interval of the initial pain-free outcome, since this study has not provided the exact definition of the initial pain-free outcome. Additionally, this study reported a higher rate of pain relief for SRS compared to PBC at the last follow-up. This finding may be due to the fact that only the actuarial probability of pain relief was provided, without including a Kaplan-Meier plot or details about censored cases and lost-to-follow-up participants. Furthermore, there was no clear definition of what constituted absolute pain relief. These findings suggest that while overall pain relief rates may be similar, the duration of relief could differ, potentially masked by variations in follow-up periods or outcome definitions in our analysis.

### Retreatment and recurrence

An important aspect of TN management is recurrence and the need for retreatment. Our study revealed SRS to be associated with a significantly lower retreatment rate compared to rhizotomy. There was also a trend towards a lower recurrence rate with SRS, which became significant in the non-MS subgroup. These results align with prior studies showing superior durability of SRS. Sanchez-Mejia et al. [27] concluded that patients who initially underwent SRS had lower retreatment rates compared to those receiving RFR. Holland et al. [20] reported that MS-TN patients who had rhizotomy as their initial procedure required significantly more procedures for relief compared to those who underwent SRS initially. Cheng et al. [15] also found fewer retreatments with SRS compared to rhizotomy in a mixed cohort of MS and non-MS patients. However, some studies highlight challenges with recurrence. Mohammad-Mohammadi et al. [24] noted that repeat procedures for MS-related TN generally had lower effectiveness across all modalities, indicating that recurrence is a persistent challenge. Thus, while our meta-analysis suggests a benefit for SRS in reducing retreatment and recurrence, particularly in non-MS patients, recurrence may still be a complex issue in MS-TN. However, a study by Sanchez-Mejia et al. [27] was detected as an outlier in our analysis due to a much lower rate of recurrence for SRS compared to other studies, and removing this study resulted in a non-significant result in the recurrence of SRS compared to rhizotomy. This study’s retrospective single-institution design may lead to selection bias due to a lack of clear descriptions and a subjective patient selection process, which was highlighted in the quality assessment. Furthermore, the follow-up duration was relatively short, which may have underestimated the recurrence rate. All patients treated with SRS in this study experienced facial numbness, suggesting there may be a trade-off between the lower recurrence rate and a higher incidence of facial numbness. Moreover, the publication bias in the recurrence meta-analysis was significant. After applying trim-and-fill, the difference in recurrence rates became insignificant, indicating that the lower recurrence rate of SRS compared to rhizotomy might be due to insufficient studies showing negative effects for SRS, highlighting the need for more research studies with larger sample sizes and rigorous design. These key differences should be considered when making clinical decisions.

### Complications

Complication profiles of SRS and rhizotomy are important aspects of treatment planning when managing a patient with TN. Common complications of SRS when treating TN include facial sensory disturbances, with 30 ;35% of patients experiencing hypoesthesia or numbness, though severe “bothersome” numbness occurs in approximately 10% of cases [64, 65]. Dysesthesia is rare, and anesthesia dolorosa, a debilitating pain in a numb area, is exceedingly uncommon. Radiation-specific risks, such as brainstem edema or necrosis, are rare but more likely with repeat procedures due to cumulative radiation exposure. Notably, SRS avoids procedural risks like infection or CSF leak. Similarly, in the case of rhizotomy, moderate-to-severe hypesthesia is the most common complication, occurring in 61% of patients. Dysesthesia occurs in 10 ;20%, and anesthesia dolorosa is observed in 0.6 ;2.4% [10, 66]. Additionally, rare but serious risks such as carotid-cavernous fistula or meningitis may arise from needle misplacement or dural penetration.

In terms of comparison, overall facial numbness rates were lower in SRS compared to rhizotomy. Similarly, while the overall complication rate was not significantly different, the MS subgroup exhibited a significantly lower overall complication rate with SRS. These findings are supported by Lee et al. [22], who found that rhizotomy resulted in more paresthesia compared to SRS in MS-TN. Holland et al. [20] reported comparable initial rates of facial numbness between SRS and rhizotomy for MS-TN, but Lee et al. [22] suggested that rhizotomy led to higher rates of sensory changes following the final treatment, possibly due to repeated procedures. Our meta-analysis provides a comparative overview across various percutaneous rhizotomy methods, but it focuses on overall rates and facial numbness specifically, which may not fully capture differences in the type or severity of complications between SRS and rhizotomy. Moreover, some discrepancies were observed in our analysis regarding facial numbness, which may be due to different thresholds and small sample sizes.

Rhizotomy provides quick relief, suitable for acute pain, but has shorter pain-free periods, higher retreatment rates, and more sensory complications, especially in MS patients. SRS has a delayed onset but lasts longer with fewer retreatments, lower facial numbness, and fewer overall complications in MS patients. Both procedures are similarly effective in pain relief. Notably, percutaneous Rhizotomy techniques tend to be less expensive than SRS, which could play an important role in decision-making [8]. However, the higher rates of recurrences and retreatments associated with Rhizotomy could shift the overall cost-effectiveness in favor of SRS, which should be considered alongside other relevant factors. This highlights the importance of a shared decision-making process between the patients and healthcare providers that considers patient priorities, clinical context, and the individual’s risk tolerance to ensure the best treatment choice [8].

### Limitations

Our meta-analysis has several limitations that should be considered; differences among the included studies in patient selection criteria, specific rhizotomy techniques, SRS protocols, follow-up durations, MS subtypes, and outcome reporting may have affected our pooled estimates and are potential sources of heterogeneity that could not be addressed in our quantitative analysis, but should be considered in clinical interpretation. Differences in how studies defined pain outcomes, such as “pain relief” and “pain-free” outcomes, as well as complications like “facial numbness,” for example, using different thresholds or scales, could impact comparability and are an important source of heterogeneity. Some studies included outcomes from initial procedures, while others incorporated repeat procedures, which may have distinct efficacy and complication profiles, potentially confounding our findings. Classifying PBC, RFR, and PRGR together as one “rhizotomy” group could mask the distinctions among these methods when analyzed against SRS. We tried to address this by qualitative description in discussion and tables, while a quantitative assessment was not possible due to a lack of sufficient suitable data. These factors, including differences, could benefit from more specific comparisons. Future research could benefit from more specific comparisons. The availability of long-term follow-up data was limited, and reasons for retreatment were not consistently reported. Understanding these reasons could elucidate mechanisms of failure and guide clinical decisions.

## Conclusion

This systematic review and meta-analysis reveal that SRS and rhizotomy are both effective options for managing TN, providing similar levels of overall pain relief. Rhizotomy stands out for its ability to deliver more rapid pain relief, making it suitable for patients needing quick results. Conversely, SRS offers greater long-term benefits, reducing the need for repeat treatments and the likelihood of pain returning, bolder in patients without MS. It also lowers the chances of facial numbness, with this advantage being more pronounced in those with MS. While the overall risk of complications is comparable between the two approaches, SRS may provide a safer profile for patients with MS. These conclusions are limited by differences across studies, inconsistent definitions of outcomes, and reliance on retrospective data, highlighting the need for future prospective research with consistent standards and extended follow-up. Treatment decisions should be tailored to each patient, weighing the urgency of pain relief against long-term effectiveness and potential side effects while considering factors like MS status, overall health, and treatment history.

## Data Availability

The data supporting this study’s findings are available from the corresponding author upon reasonable request.
